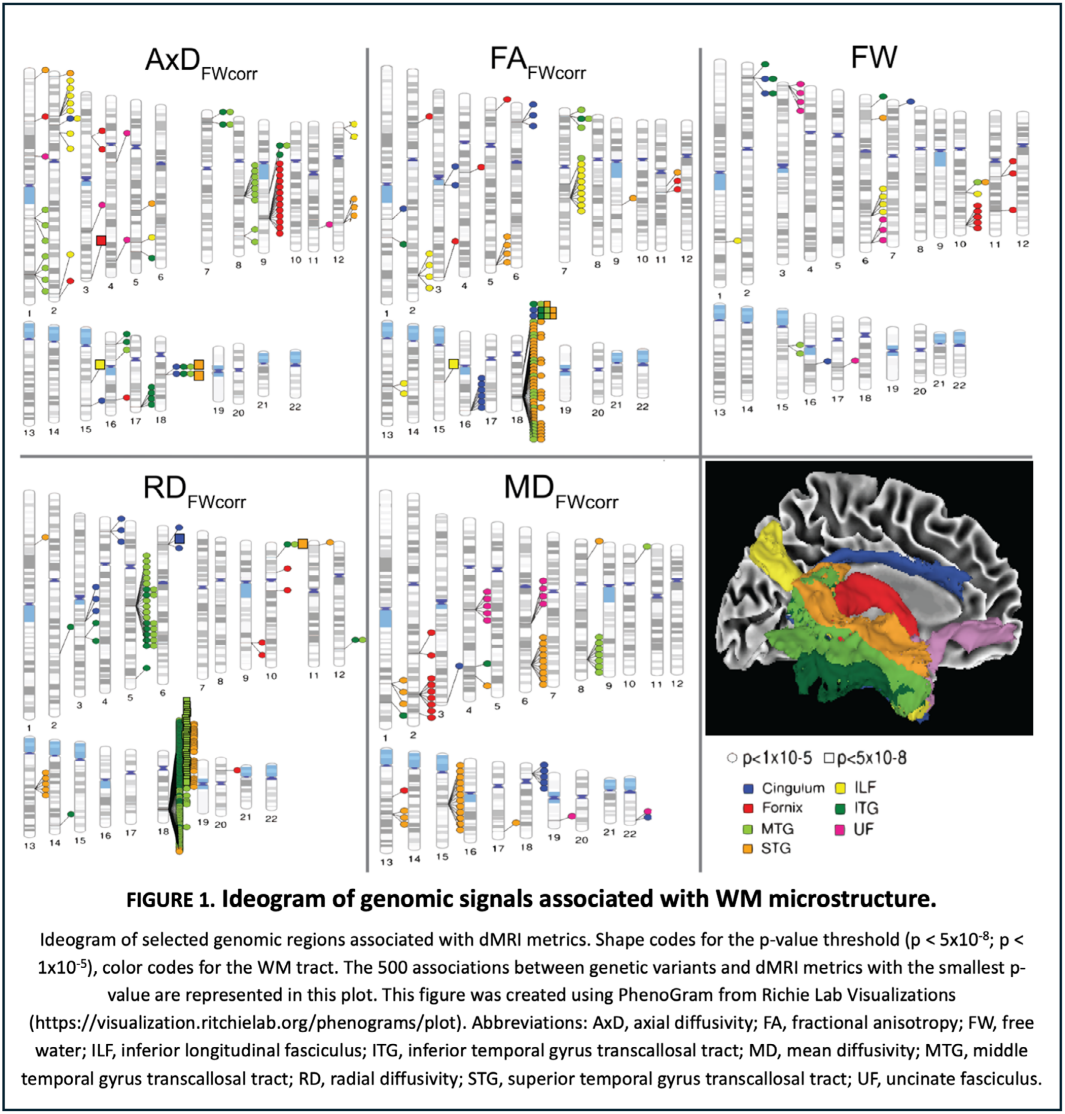# Genetic architecture of the limbic white matter microstructure in aging and Alzheimer's Disease

**DOI:** 10.1002/alz70856_098641

**Published:** 2025-12-24

**Authors:** Anna S Lorenz, Aditi Sathe, Yisu Yang, Alaina Durant, Yiyang Wu, Michael E Kim, Chenyu Gao, Nancy R Newlin, Karthik Ramadass, Praitayini Kanakaraj, Nazirah Mohd Khairi, Zhiyuan Li, Tianyuan Yao, Yuankai Huo, Logan Dumitrescu, Niranjana Shashikumar, Kimberly R. Pechman, Shannon Risacher, Lori L Beason‐Held, Yang An, Konstantinos Arfanakis, Guray Erus, Christos Davatzikos, Mohamad Habes, Di Wang, Duygu Tosun, Arthur W. Toga, Paul M. Thompson, Elizabeth C. Mormino, Panpan Zhang, Kurt Schilling, Marilyn S. S. Albert, Walter W. Kukull, Sarah Biber, Bennett A. Landman, Sterling C Johnson, Barbara B. Bendlin, Julie A Schneider, Lisa L. Barnes, David A. A. Bennett, Angela L. Jefferson, Susan M. Resnick, Andrew J. Saykin, Timothy J. Hohman, Derek Archer, Alzheimer's Disease Neuroimaging Initiative (ADNI), The BIOCARD Study Team, The Alzheimer's Disease Sequencing Project (ADSP)

**Affiliations:** ^1^ Vanderbilt Memory and Alzheimer's Center, Nashville, TN, USA; ^2^ Vanderbilt Memory & Alzheimer's Center, Vanderbilt University Medical Center, Nashville, TN, USA; ^3^ Vanderbilt Memory and Alzheimer's Center, Vanderbilt University School of Medicine, Nashville, TN, USA; ^4^ Vanderbilt University Medical Center, Nashville, TN, USA; ^5^ Vanderbilt Memory and Alzheimer's Center, Vanderbilt University Medical Center, Nashville, TN, USA; ^6^ Vanderbilt University, Nashville, TN, USA; ^7^ Vanderbilt Memory and Alzheimer's Center, Vanderbilt University School of Medicine, Nashville, TN, USA; ^8^ Department of Radiology and Imaging Sciences, Indiana University School of Medicine, Indianapolis, IN, USA; ^9^ Indiana Alzheimer's Disease Research Center, Indianapolis, IN, USA; ^10^ Laboratory of Behavioral Neuroscience, National Institute on Aging, Intramural Research Program, Baltimore, MD, USA; ^11^ Brain Aging and Behavior Section, National Institute on Aging, NIH, Baltimore, MD, USA; ^12^ Rush Alzheimer's Disease Center, Rush University Medical Center, Chicago, IL, USA; ^13^ Department of Diagnostic Radiology and Nuclear Medicine, Rush UniversityRush University Medical Center, Chicago, IL, USA; ^14^ Artificial Intelligence in Biomedical Imaging Laboratory, Perelman School of Medicine, University of Pennsylvania, Philadelphia, PA, USA; ^15^ Center for AI and Data Science for Integrated Diagnostics, University of Pennsylvania, Philadelphia, PA, USA; ^16^ University of Texas Health San Antonio, San Antonio, TX, USA; ^17^ UT Health San Antonio, San Antonio, TX, USA; ^18^ University of California, San Francisco, San Francisco, CA, USA; ^19^ Laboratory of Neuro Imaging, Stevens Neuroimaging and Informatics Institute, Keck School of Medicine, University of Southern California, Los Angeles, CA, USA; ^20^ Imaging Genetics Center, Mark and Mary Stevens Neuroimaging and Informatics Institute, Keck School of Medicine, University of Southern California, Marina del Rey, CA, USA; ^21^ Department of Neurology and Neurological Sciences, Stanford University School of Medicine, Stanford, CA, USA; ^22^ Department of Biostatistics, Vanderbilt University Medical Center, Nashville, TN, USA; ^23^ Department of Radiology & Radiological Sciences, Vanderbilt University Medical Center, Nashville, TN, USA; ^24^ Johns Hopkins University School of Medicine, Baltimore, MD, USA; ^25^ National Alzheimer's Coordinating Center, University of Washington, Seattle, WA, USA; ^26^ Wisconsin Alzheimer's Institute, University of Wisconsin‐Madison School of Medicine and Public Health, Madison, WI, USA; ^27^ Wisconsin Alzheimer's Disease Research Center, University of Wisconsin‐Madison, School of Medicine and Public Health, Madison, WI, USA; ^28^ Rush University, Chicago, IL, USA; ^29^ National Institute on Aging, National Institutes of Health, Baltimore, MD, USA; ^30^ Indiana University School of Medicine, Indianapolis, IN, USA

## Abstract

**Background:**

Limbic white matter (WM) abnormalities are strongly elevated along the Alzheimer's Disease (AD) diagnostic continuum, but the underlying biological mechanisms remain unclear. This study aims to conduct a large‐scale genetic analysis of WM microstructure in older adults.

**Method:**

WM was assessed in seven limbic tracts, including the cingulum, fornix, inferior longitudinal fasciculus (ILF), uncinate fasciculus (UF), and transcallosal tracts of the inferior, middle, and superior temporal gyri (ITG, MTG, STG) using advanced diffusion MRI metrics corrected for free‐water (FW) (fractional anisotropy [FA_FWcorr_], axial diffusivity [AxD_FWcorr_], mean diffusivity [MD_FWcorr_], radial diffusivity [RD_FWcorr_]). Genetic associations with WM microstructure were investigated using harmonized data from seven aging cohorts, comprising 2,614 non‐Hispanic white older adults (mean age = 73.66 ± 9.76; 42.65% male), through SNP‐heritability estimation, genome‐wide association studies (GWAS), and post‐GWAS analyses (genetic correlation, gene‐level, and pathway analysis). Bulk RNA‐seq brain data were used to evaluate the relationship between expression of genes identified in the GWAS with cognition and AD pathologies.

**Result:**

WM microstructure is heritable, with 16 of 35 metrics exhibiting estimates between 0.26 and 0.60 (p_FDR_<0.05). Genome‐wide associations (*p* <5×10^−8^) were observed for fornix AxD_FWcorr_ (chr3, rs78407651), ILF FA_FWcorr_ (chr15, rs8026709) and AxD_FWcorr_ (chr15, rs8026709), STG RD_FWcorr_ (chr10, rs11542181), and cingulum RD_FWcorr_ (chr6, rs56017587). A locus with 38 genome‐wide significant SNPs (chr18, rs12959877) was associated with FA_FWcorr_ and RD_FWcorr_ (Figure 1). These SNPs are eQTLs for *CDH19*, a gene highly expressed in oligodendrocytes with a role in cell adhesion. Among the genes identified in the GWAS, *RORA*, *FAM107* and *KC6* expression in brain tissues was linked to cognitive decline and AD pathologies (p_FDR_<.05). Gene‐level analysis highlighted *SERPINA12* (z=4.60, p_FDR_=.03), a gene implicated in type 2 diabetes and atherosclerosis. Pathway analysis revealed associations with insulin, immune response, and neurotrophic signaling. Genetic correlations were identified with lipid profiles, cardiovascular traits, and neuropsychiatric conditions (p_FDR_<.05).

**Conclusion:**

This study identified genetic factors related to cognition, vascular health, and inflammation as contributors to WM microstructure changes in aging and AD. These findings open avenues for future research on AD's molecular mechanisms and therapeutic targets for improving vascular and metabolic health in aging populations.